# Introductory Lecture to the Winter Course of Instruction in the Philadelphia College of Medicine

**Published:** 1850-12

**Authors:** 


*Introductory Lecture to the Winter Course of Instruction in the Philadelphia College of Medicine. Delivered, Oct.* 14, 1850. By JAMES M'-CLINTOCK, M. D., Professor of Surgery and Anatomy. Published by the Class.

Introductory discourses, thus far, since the "flowering season" has commenced, have been like angel's visits in that they are few and far between. The lecture by Professor M'Clintock, in fact, is the only one that has, as yet, reached us. Usually the crop has been so abundant that we have despaired of being able to render to all parties even handed justice, and, therefore, to avoid apparent injustice by an invidious discrimination, we have been obliged to serve all alike by simply acknowledging the courtesy which did not overlook us in the distribution of copies.

As the solitary representative of this species of medical periodical liter. ture, we will devote some space to the discourse just mentioned, not wishing, however, that this course shall be taken as a precedent for future action, provided our editorial table becomes crowded, as usual, with similar favors.

The subject of Prof. M'Clintock's lecture will appear from the following extract:—

In choosing a topic for discussion to-day, I have thought it best, instead of dwelling upon broad generalities, to select a single line of remark, upon a subject of wide interest however to you and to our whole profession. My purpose will be to correct a misapprehension which prevails in many quarters in regard to the scope of Surgery, and the relation which subsists between the medical practice of the Surgeon and that of the Physician. Many persons out of our profession imagine that the duties of the Surgeon are confined to the management of external diseases and the performance of manual operations. It readily follows from this notion, wherever it is imbibed, that a man may be a skilful surgeon without being a capable physician; and from this again it is not difficult, for the common mind, by one of those leaps of induction to which it is so remarkably liable, to infer that the skilful surgeon is not *likely* to be a good physician. This, gentlemen, is the misapprehension which I now propose to correct.

Having thus unfolded the topic of the discourse, he next develops the position which it is the object of the remainder of the discourse to advocate and illustrate. We quote as follows :—

It might suffice for my present purpose to show you, from the very nature of surgery itself, that no man is qualified to practice it without a full acquaintance with the other branches of medicine ; but I shall go further, and show you on the other hand, that the ordinary routine of medical practice so often requires the aid of that branch of the profession which is technically called surgery, that no man is competent to discharge the duties of the physician fully, who is not at the same time familiar, to a certain extent at least, with both the principles and practice of surgery. It will follow, then, gentlemen, necessarily, that, as a general rule, the *good* surgeon is the *best* physician.

The postulates assumed in the foregoing paragraph are these:—a knowledge of medicine proper, is necessary to the surgeon. A knowledge of surgery is necessary to the physician. Ergo, the "*good* surgeon is the *best* physician."

We cannot entirely concur in the logic by which the lecturer reaches the above conclusion. Since he admits that a knowledge of medicine is necessary to the surgeon, as well as *vice versa,* by the same method of reasoning, we may say that the *good* physician is the *best* surgeon. The rule works equally well in both ways. The author is a teacher of surgery, and views the subject as it may be gratifying to a surgeon to look at it. His colleague in the chair of medicine, however, we trow, will hardly hold up to the class of pupils attending the Philadelphia College, the pre-eminent importance of close attention to the lectures on surgery, as the best means of acquiring a knowledge of the branches which it is his prerogative to teach.

The converse of the position taken by Prof. M'Clintock would, in fact, be much nearer the truth. The better acquainted a surgeon is with the principles of medicine, the better qualified is he to practice surgery; because in every thing relating to the treatment of external diseases, save what concerns the use of the knife, these principles are involved, not less than in the duties of the physician. And since, according to the maxim of modern conservative surgery, *cutting* is only an alternative when *curing* fails, knowledge of the latter is much the more important of the two. We should vastly prefer to trust life and limb in the hands of the surgeon most competent to cure, than of one whose chief aim was to excel as a dexterous operator. The cultivation and application of therapeutical principles in the treatment of surgical affections is the best safeguard against pruriency in the use of the scalpel.

The ground taken by Prof. M., is not only untenable, and absurd, but, if heeded by medical students, could not fail to prejudice their interests, by encouraging them to undervalue instruction in the diagnosis, treatment, etc., of medical, as distinguished from surgical diseases.

As the lecturer justly remarks, Surgery and Medicine cannot be absolutely separated. They are different branches, with the same trunk and roots. The good surgeon must be a good physician we admit, but it is a *non sequitur* that the good physician must be a surgeon, still less that ability as a physician is commensurate with excellence as a surgeon. The latter is a preposterous conclusion, which we can hardly conceive of any person seriously entertaining, who had devoted much study to the subjects involved in medical practice.

Prof. M. cites the examples of several distinguished surgeons who were successful as medical practitioners. This we admit, but we deny that their *real* success, as practitioners of medicine, depended on their skill as surgeons. The eclat of surgical eminence has sometimes favored the acquisition of medical practice, owing to an impression, somewhat current with the public, that because a person operates adroitly, he can therefore cure diseases better than those who devote exclusive attention to the latter. We are sorry to see this popular error formally promulgated from a surgical chair in a medical college.

